# The mutant mouse resource and research center (MMRRC) consortium: the US-based public mouse repository system

**DOI:** 10.1007/s00335-024-10070-3

**Published:** 2024-09-20

**Authors:** Yuksel Agca, James Amos-Landgraf, Renee Araiza, Jennifer Brennan, Charisse Carlson, Dominic Ciavatta, Dave Clary, Craig Franklin, Ian Korf, Cathleen Lutz, Terry Magnuson, Fernando Pardo-Manuel de Villena, Oleg Mirochnitchenko, Samit Patel, Dan Port, Laura Reinholdt, K. C. Kent Lloyd

**Affiliations:** 1 Department of Veterinary Pathobiology, College of Veterinary Medicine, University of Missouri, Columbia, MO USA; 2 Department of Molecular and Cellular Biology, College of Biological Sciences and Bioinformatics Core, Genome Center, University of California, Davis, CA USA; 3 Mouse Biology Program, University of California, Davis, CA USA; 4 Department of Genetics and Lineberger Comprehensive Cancer Center, University of North Carolina-Chapel Hill, Chapel Hill, NC USA; 5 The Jackson Laboratory, Bar Harbor, Maine USA; 6 Division of Comparative Medicine, Office of Research Infrastructure Programs, Division of Program Coordination, Planning, and Strategic Initiatives, Office of the Director, National Institutes of Health, Bethesda, USA; 7 Department of Surgery, School of Medicine, University of California, Davis, CA USA

**Keywords:** Mouse, Repository, Genetics, Cryopreservation, Phenotyping, Disease model

## Abstract

Now in its 25th year, the Mutant Mouse Resource and Research Center (MMRRC) consortium continues to serve the United States and international biomedical scientific community as a public repository and distribution archive of laboratory mouse models of human disease for research. Supported by the National Institutes of Health (NIH), the MMRRC consists of 4 regionally distributed and dedicated vivaria, offices, and specialized laboratory facilities and an Informatics Coordination and Service Center (ICSC). The overarching purpose of the MMRRC is to facilitate groundbreaking biomedical research by offering an extensive repertoire of mutant mice that are essential for advancing the understanding of human physiology and disease. The function of the MMRRC is to identify, acquire, evaluate, characterize, cryopreserve, and distribute mutant mouse strains to qualified biomedical investigators around the nation and the globe. Mouse strains accepted from the research community are held to the highest scientific standards to optimize reproducibility and enhance scientific rigor and transparency. All submitted strains are thoroughly reviewed, documented, and validated using extensive scientific quality control measures. In addition, the MMRRC conducts resource-related research on cryopreservation, mouse genetics, environmental conditions, and other topics that enhance operations of the MMRRC. Today, the MMRRC maintains an archive of mice, cryopreserved embryos and sperm, embryonic stem (ES) cell lines, and murine hybridomas for nearly 65,000 alleles. Since its inception, the MMRRC has fulfilled more than 20,000 orders from 13,651 scientists at 8441 institutions worldwide. The MMRRC also provides numerous services to assist researchers, including scientific consultation, technical assistance, genetic assays, microbiome analysis, analytical phenotyping, pathology, cryorecovery, husbandry, breeding and colony management, infectious disease surveillance, and disease modeling. The ICSC coordinates MMRRC operations, interacts with researchers, and manages the website (mmrrc.org) and online catalogue. Researchers benefit from an expansive list of well-defined mouse models of disease that meet the highest scientific standards while submitting investigators benefit by having their mouse strains cryopreserved, protected, and distributed in compliance with NIH policies.

## Introduction

**Fig. 1:**
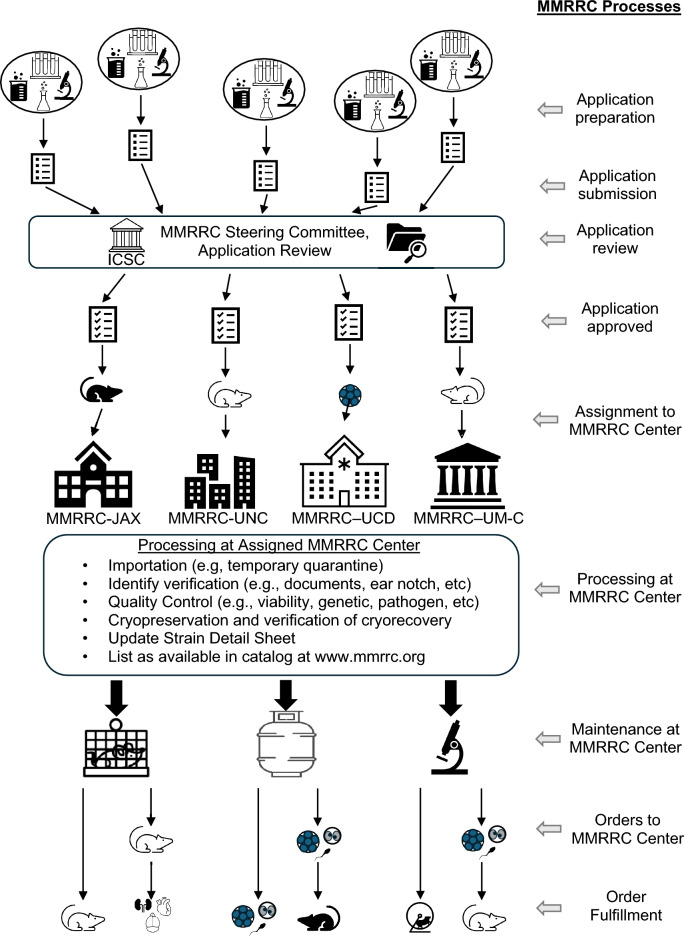
Diagram representing submission of mouse strains, ES cell lines, and hybridomas to the MMRRC. Applications are reviewed by the MMRRC Steering Committee. Once accepted, materials are assigned to an MMRRC Center which conducts multiple processes to make it available for distribution. Orders for mice, cryopreserved materials, cryorecovery of mice from the cryoarchive, mice derived by blastocyst injection of ES cells, and tissues harvested from mice are fulfilled by the assigned MMRRC Center

The Mutant Mouse Resource and Research Center (MMRRC) is the nation’s pre-eminent public mutant mouse archive and distribution repository system (RRID:SCR_002953). Established in 1999 by the NIH to ensure the preservation, dissemination, validation, and development of scientifically valuable mutant mouse strains and data produced by individual research scientists, the MMRRC consists of a US-based network of regional archive and distribution repositories located at the University of North Carolina—Chapel Hill (RRID:SCR_016449), University of Missouri—Columbia (RRID:SCR_016447), The Jackson Laboratory(RRID:SCR_016446), and the University of California Davis(RRID:SCR_016448). In addition, an Informatics Coordination and Service Center (ICSC), also at the University of California Davis, coordinates MMRRC operations, facilitates interactions with researchers, manages the website (mmrrc.org), and maintains an online catalogue. The MMRRC accepts and ensures access to transgenic, knockout, CRISPR-edited, and other genetically altered mutant mice and related biomaterials, services, and technologies to biomedical researchers. The MMRRC imports, verifies, maintains, characterizes, and distributes mice, gene-targeted murine embryonic stem (ES) cell lines, murine hybridomas, tissues, and frozen embryos and germplasm of genetically unique mice to research scientists upon request (Fig. 1). MMRRC institutions also provide services and procedures to create, modify, recover, manage, and study genetically altered mice and cryomaterials for applied and translational research in numerous scientific areas, including cancer, neurodegenerative, neurosensory, metabolic, musculoskeletal, reproductive, developmental, genetic, and other diseases, disorders, and syndromes. Further, MMRRC scientists are funded to conduct resource-related research and to develop and refine technologies that capitalize on the power of mouse genetics for biomedical research and enhance the function and services of the MMRRC resource. By submitting their mouse models for deposition into and distribution by the MMRRC, NIH-funded investigators fulfill their obligations under NIH data and resource sharing policies. In return, the MMRRC ensures the preservation, protection, quality control, viability, and accessibility of these resources to the biomedical research community. Further, while based in the United States, the MMRRC is available to the international community for researchers from around the world interested in submitting and sharing their scientifically valuable mouse strains.

## Origin

In the 1990s, an expansion of the repertoire and use of mouse models of human disease was underway (Davisson and Taft 2006, Gurumurthy and Lloyd 2019). A myriad of technologies, including N-ethyl-N-nitrosourea (ENU) mutagenesis, homologous recombination in ES cells, transgenic, gene trap, conditional targeting (e.g., Cre, Flp), transposons, and programmable endonucleases (e.g., CRISPR/Cas9) precipitated rapid growth in the number and complexity of engineered mouse models being developed for research. As the few commercial repositories became overwhelmed, direct sharing of mouse models among individual investigators ensued, often without regard for proper genetic quality control or testing for adventitious pathogens. This led to several concerning consequences, including diminished experimental rigor and reproducibility, loss of equitable access and transparency, lack of appropriate attribution, escalating costs, and more. These and other challenges became widespread, negatively impacting the reliability and reproducibility of scientific results using mice (and other animals), especially in translational preclinical studies (Kilkenny et al 2009). This prompted the development of a coordinated system that would enable fair, professional, and sustainable archive, curation, and distribution services for the research community using mutant mice.

In response, NIH convened a panel of internationally renowned scientists in 1998 to draft recommendations for facilitating access to and economizing mutant mouse resources. These recommendations included the creation of a US consortium of regionally positioned archive and distribution repositories operated by professional technical experts with experience in mouse genetics, cryopreservation, husbandry, and veterinary care, supervised by scientists conducting resource-related research. Now funded and overseen by the NIH Office of Research Infrastructure Programs [ORIP], Division of Program Coordination, Planning, and Strategic Initiatives [DPCPSI], Office of the Director, the MMRRC was officially launched as the nation’s only publicly accessible mutant mouse archive and distribution repository system (Grieder 2002).

## Mission and goals

The overarching purpose of the MMRRC is to facilitate broad, equitable, ready, and controlled access to mutant mice and mouse resources under rigorous quality control standards (Battey et al 1999). To do so, the MMRRC provides research resources that help optimize and enhance scientific rigor, transparency, and experimental reproducibility of biomedical research using genetically altered mice. The MMRRC aims to acquire and provide mutant mice, sperm, embryos, ES cell lines, and hybridomas to qualified biomedical researchers at research centers, academic institutions, for-profit organizations, the NIH, and other federal agencies. To successfully fulfill this mission, the MMRRC (1) applies innovative approaches to interact with the research community, (2) utilizes innovative acquisition, characterization, validation, verification, maintenance, preservation, and husbandry techniques and technologies applicable to mice, embryos, germplasm, and ES cells lines, and hybridomas, (3) coordinates activities through consortium-wide operational and performance metrics, joint goals, and strategies to address the evolving needs of the biomedical research community, (4) raises awareness and community outreach at scientific meetings, (5) solicits mouse models from researchers prior to and after publication, and (6) establishes and implements disaster and recovery plans to prevent, preserve, and protect against loss of biological and data resources in the event of a catastrophic disaster (*e.g.,* hurricane, power loss, fire, flood) or cyberattack.

## Infrastructure and organization

Institutions that host the distribution repositories for the MMRRC have the scientific expertise, experience, capability, and capacity to manage mice and mouse resources safely, reliably, and securely. These characteristics ensure the viability, genetic identity, pathogen-free status, and long-term availability of scientifically valuable mutant mouse models for researchers. Each MMRRC Center exists in a research-intensive environment that synergizes with in-house genetic and programmatic resources and expertise that reinforces the MMRRC mission (Table 1). Centers are led by a scientific expert in fields (genetics, cryopreservation, reproduction, etc.) related to the use of genetically-altered mice in biomedical research. Each Center also has a administrator who oversees a team of technical experts, animal handlers, logistics managers, business personnel, and other staff who maintain daily office, laboratory, and vivarium operations. Together they engage in close coordination and working relationships as a US-based consortium of individual, regionally distributed Centers with dedicated infrastructure (including barrier housing and microbial quality control), cryopreservation and reanimation capabilities, proven standard operating procedures (SOPs), surveillance protocols for infectious pathogens and environmental contaminants, and professional, scientific, and technical expertise in quality control and other activities to maintain the genetic integrity and specific pathogen-free status of mice, embryos, germplasm, ES cell lines, and hybridomas. The Centers all have robust animal infrastructure (vivaria, technicians, veterinary and laboratory support), mouse husbandry and care experience, and infectious and genetic quality control and assurance standards, and policies in place to ensure research reproducibility and integrity. These characteristics are fundamental expectations of centralized mouse repository systems worldwide (Donahue et al. 2012).

**Table 1:** Location and web addresses of (current) Centers in the MMRRC Consortium

Host institution (*year established*)	Web address
University of Missouri, Columbia (*1999*)	https://mu-mmrrc.com
University of California, Davis (*1999*)	https://mmrrc.ucdavis.edu
University of North Carolina, Chapel Hill (*1999*)	https://med.unc.edu/mmrrc
The Jackson Laboratory (*2010*)	https://jax.org/research-and-faculty/resources/mutant-mouse-resource-research-center

## Best practices

The MMRRC has developed a common set of SOPs that govern general operations for importation and distribution of mutant mice, cryopreservation, maintenance, and cryorecovery, pathogen, health and genetics surveillance and monitoring of archived strains, curation and presentation of data and information, and community outreach (Table 2).

**Table 2:** MMRRC consortium best practices

Participating MMRRC Centers observe best practices in the following activities:	▪ importation, rederivation, maintenance (e.g., husbandry, care, and welfare), and distribution of mice and materials
	▪ cryopreservation and recovery, pathogen surveillance, and health monitoring
	▪ information technology, database management, and website and online services
	▪ communications, outreach, and education

Centers harmonize internal SOPs within the MMRRC Consortium while maximizing individual efficiencies. The Centers also serve as *centers of excellence* in mouse genetics, biology, and phenotyping, thereby facilitating and extending the validity, reproducibility, and scientific value of mouse models in their care. In some cases, they have earned “approved vendor” status from institutions of requesting investigators, facilitating direct import of mice into research colonies, which bypasses costly delays associated with importation quarantines and rederivation. Each Center director serves on the MMRRC Coordinating Committee (CC), which holds monthly meetings to address operational coordination, functional organization, and management activities. The CC receives advice and recommendations on MMRRC operations, policies, outreach, research, and scientific merit of submitted strains from a five-member External Advisory Panel of outside investigators with expertise in mouse biology and phenotyping, resource function and operations, information technology, and the biotechnology industry. In addition, the CC uses instant “check-out” surveys and solicits input from MMRRC users on satisfaction, quality, services, and other needs to help improve the effectiveness of the MMRRC to meet the needs of researchers.

## Submission of mouse strains

Any scientist from anywhere in the world who has developed a mutant mouse strain that is applicable for biomedical research is welcome to complete a submission application form that can be found on the MMRRC website (https://www.mmrrc.org/submission). The form requests information about the submitting scientist and strain developer, the genetic background, alteration(s) and phenotype(s) of the strain, mouse breeding and management strategy, potential research applications, publications, and other information and data that would be informative and useful for investigators interested in potentially ordering the strain for use in their research program. Researchers can submit their mouse models to the MMRRC in one of three ways (Table 3). Most submissions are *Type 1*, which are unsolicited, spontaneous, and voluntary applications tendered by the originator of the mouse strain or their designee. At monthly meetings of the CC, submission applications are reviewed to determine whether they fulfill the six criteria necessary for a mouse strain to be accepted into the MMRRC (Table 4). Although not required, a scientific publication describing the creation and study of the submitted mouse strain adds valuable data to facilitate review.

**Table 3:** Types of mouse submissions to the MMRRC program

MMRRC submission type	Description
Type 1	Individual investigators
Type 2	NIH program officer-sanctioned
Type 3	Contracted to individual center

**Table 4:** Review criteria for mouse strains accepted into the MMRRC

1. Created using sound scientific methods
2. Distinguishable genotypically and/or phenotypically from other mouse strains
3. Exhibits an identifiable phenotype
4. Exists at no other distribution Center
5. Available for distribution to the scientific research community
6. Controls are readily available or can be provided by the submitting investigator

On the other hand, *Type 2* and *Type 3* submissions do not undergo formal review by the CC. Instead, the MMRRC provides NIH categorical institutes with a limited number of *Type 2* submission “slots” pre-authorized for acceptance of mice generated by their intramurally-funded scientists. *Type 3* submissions are funded by the submitter and are primarily acquired as thematic collections, such as mouse models of Alzheimer’s diseases (Esquerda-Canals et al 2017), Collaborative Cross strains (Threadgill et al 2011), and GENSAT (Condie 2016), or specialized libraries that were produced by large, often NIH-funded consortiums such as KOMP (Brown and Moore 2012), commercial organizations, and private research entities. Some of these collections are submitted and made available for distribution as gene-trap or gene-targeted ES cells, several of which have been converted into mice and are available as cryopreserved germplasm.

For all accepted and pre-authorized mouse strains, the only costs to the submitting investigator are those associated with shipping mice to the assigned Center. Otherwise, the assigned Center bears all costs for importation, rederivation, quality control testing, maintenance, and archiving using their awarded NIH funds. Once imported into one of the fore-mentioned Centers, tissue samples are taken for DNA extraction to confirm the genotype of the mutant allele and fidelity of the genetic background, fecal pellets are harvested (or collected earlier by the submitting investigator before shipping mice) for microbiome analysis (Ericsson and Franklin 2021), mice are rederived and bred if necessary to generate live mouse colonies, and sperm and/or embryos are cryo-archived. Once fully curated and after confirmation of genotype and genetic background, establishment of a cryopreserved archive and verification of successful cryorecovery, determination of pathogen-free status, and exclusion of unintended or contaminating DNA elements (e.g., Cre), mice are listed as available for ordering through the online catalog. This verification process takes several weeks and ensures the accuracy and reliability of the submitted strain. Proceeds derived from modest fees charged for mice requested by investigators provide program income to support costs associated with distribution (e.g., cryorecovery supplies, housing, breeding, animal care, technical effort, genotyping, shipping, and handling, etc.) incurred by the MMRRC.

## Availability of mouse resources

The multi-tiered submission application system has enabled the MMRRC to acquire and make thousands of mutant mouse alleles available to the research community that otherwise might not be. Several large collections from commercial entities have been acquired by the MMRRC and are available for distribution to the academic research community. The growth in the number of holdings in the MMRRC Program has increased over the last 25 years to a total of 64,796 unique mutant alleles (Table 5), making it one of the largest public repositories of mutant mouse strains in the world today. These strains are available in one or more forms as live mice, cryopreserved germplasm, pluripotent ES cell lines, hybridomas, and/or tissues. MMRRC mouse models are useful for research in many disease categories including oncology, cardiology, immunology, neurology, developmental biology, and to study specific syndromes and diseases. Investigators interested in submitting their mouse strain to the MMRRC Program can submit information online through the MMRRC website (https://www.mmrrc.org/submission/strain_submission_terms.php), contact the Strain Acquisition Coordinator at the ICSC, or contact the Import Coordinator at any of the Centers for information on strains, assistance with finding and selecting strains from the catalog, and instructions for how to complete submission and request forms.

**Table 5:** Mutant mouse strains accepted into the MMRRC Program

Deposition type	Cumulative (2000–2025)
Type 1: Investigator initiated, MMRRC supported	2437
Type 2: NIH initiated, MMRRC supported	295
Type 3: Investigator initiated and supported	62,064
Totals	64,796

## Distribution of mouse strains

The number of researchers using the MMRRC has steadily increased over the last 25 years. Despite the temporary drop in usage resulting from the impact of the global SARS-CoV-2 pandemic on research activity and on-site laboratory work during most of 2020, this number of users has translated into a substantial number of orders received and fulfilled by the MMRRC since opening its doors. As of 2024, 13,651 investigators at 8441 institutions in more than thirty countries worldwide have placed over 20,200 orders from the MMRRC (Table 6). These orders span the gamut of breeding pairs of mice, cryopreserved embryos, sperm, ES cell lines, or hybridomas, and live mice produced from cryopreserved embryos, sperm, or ES cells. In addition, individual MMRRC Centers provide frozen and processed (e.g., formalin-fixed) tissues from mouse strains to investigators upon request. Many researchers are returning MMRRC customers who come back to request not only mouse strains but also special services from the host institution. For example, several of the individual MMRRC Centers can provide model development services to create new models if one does not already exist in the repository. In addition, MMRRC Centers distribute live mice from vivaria operating under specific pathogen-free conditions, enabling requesting Investigator’s institutions to grant “approved vendor” status to the MMRRC and expedite processing of imported mouse strains.

**Table 6:** Orders for mutant mouse strains from the MMRRC program

Metric	Cumulative 2000–2025
Orders^1^	20,225
Investigators^2^	13,651
Institutions^2^	8441

^1^can include multiple mouse strains, mice

^2^unique

With the assignment of unique Research Resource Identifiers (RRID; (https://scicrunch.org/resources) (Bandrowski and Martone 2016) to each MMRRC mouse strain, a search of relevant databases (e.g., PubMed) has identified 3122 research articles published to date using mouse strains obtained from the MMRRC, reflecting the ongoing reliance by the research community on the MMRRC as a vital public resource. This number of publications is surely underrepresented, as new articles are discovered daily.

## Improving resource quality

The MMRRC also provides several ancillary services and procedures that can be accessed by users of the MMRRC. These services include colony management and breeding, mouse assisted reproductive technologies, recovery and reanimation of cryopreserved embryos and germplasm, ES cell and microinjection, transgenic and other production (CRISPR/Cas9) approaches, genotyping, and genetic analysis, in vivo and ex vivo phenotyping, clinical diagnostics, and anatomic pathology. These and other ancillary services are of significant value to researchers who have varying levels of technical skills and resources for the handling imported frozen embryos and sperm, particularly at a time when long distance shipping of live mice is becoming more difficult and expensive. Individuals, institutions, or consortia may arrange for the acquisition, importation, cryopreservation, and distribution of individual mouse strains or collections on a fee-for-service basis from a Center. A table of available services and contact information at each Center can be found at www.mmrrc.org/about/members.php. In addition, the MMRRC provides access to a number of SOPs it uses for operations. For example, protocols detailing methods and procedures for genotyping of DNA extracted from tail tissue, colony management and mouse breeding, cryopreservation and cryorecovery, and ES cell culture, selection, and injection are available from the MMRRC-UC Davis website (mmrrc.ucdavis.edu).

One of the more recently established services is a derivative of the Mouse Universal Genotyping Array (MUGA) that was originally developed to genetically characterize the Collaborative Cross and Diversity Outbred lines of mice (Morgan et al. 2015). The MiniMUGA, an array-based high-density single nucleotide polymorphism (SNP) genetic quality control platform using over 11,000 probes to discriminate genotypically between a variety of wild-derived and laboratory-produced mouse strains, retains the genetic discrimination and robust properties as MUGA (Sigmon et al. 2020). In addition, MiniMUGA determines the parental sex chromosomes, discriminates substrains from commercial vendors, identifies SNPs diagnostic for individual laboratory strains, and detects exogenous DNA elements and constructs (e.g., neomycin, loxP, etc.) typically used for genetic modification of alleles. All mouse strains incoming to the MMRRC undergo a MiniMUGA analysis as part of the regular curation process. A Strain Detail Sheet (SDS) containing strain (gene details and genotyping protocols, genetic background, strain development, phenotyping data, recommended control mice, genetic quality control, research applications, and strain origin), colony and husbandry (health status, general appearance, breeding strategy, reproductive statistics), and ordering (availability level, conditions of distribution, fees) information on each strain is posted in the online catalog.

Similarly, the MMRRC is actively investigating the role of differing intestinal microbiota on model phenotypes and has developed tools that aid investigators in considering microbiota in model reproducibility troubleshooting and optimization (Ericsson and Franklin 2021). For example, centers may harvest fecal pellets or have them collected by submitting investigators prior to shipment. Analysis of samples collected to date have shown marked variability in the fecal microbiome of mice submitted and related studies have demonstrated that differences in microbiota can significantly modulate certain model phenotypes. These archived microbiota data are available through the SRA public database and can be used in troubleshooting situations where investigators are unable to reproduce published phenotypes. In such cases, should microbiota differences correlate with differing phenotypes, strains can be rederived onto select complex microbiota based on archived data or standardized complex microbiota that represent extremes of those seen in contemporary rodent colonies (Hart et al 2018).

## Resource-related scientific research

Innovative resource-related research is critical to enhancing MMRRC consortium operations and services. This research has improved cryopreservation and recovery of embryos (Mochida et al 2012), refinement of in vitro fertilization (IVF) and intracytoplasmic sperm injection (ICSI) (Li et al 2003, 2015), enhanced isolation, purification, and gene targeting in murine ES cells (Pettit et al. 2009), improved pathogen detection assays (Ericsson et al 2017; Agca 2017), enhanced genome editing (CRISPR/Cas9) (Modzelewski et al 2018), and the establishment of strategies by which to provide requesting investigators with mice on select complex microbiota (Hart et al 2018, Ericsson et al. 2021). In this way, the MMRRC Program is constantly adapting to the rapidly changing needs of the scientific community by mitigating negative impacts of growing mouse populations on the nation’s infrastructure. Further, electronic networking and innovative data management that enables integration of data and information from multiple sites, automated tools, curation, attention to cybersecurity, and quality control have been incorporated by the ICSC into the MMRRC Consortium. Centers provide training and professional expertise to maximize their impact and access by the scientific community.

A particularly important research area of the MMRRC involves studies to enhance and improve cryopreservation and reanimation of mice from cryopreserved sperm (Li et al 2014; Agca and Agca 2021; Gerb et al 2022; Wuri et al 2019). With improvements in methodology, sperm cryopreservation has become the primary approach to creating a cryoarchive of mouse strains maintained by the MMRRC. While sperm cryopreservation is efficient and cost effective, cryorecovery success rates must be proven sufficiently robust and reproducible between the MMRRC and individual laboratories since the MMRRC allows investigators to order cryopreserved sperm in addition to live mice.

## Scientific, customer, and technical support

A primary goal of the MMRRC is to present a single, unified online presence to best serve its users, both submitters and requesters. The ICSC coordinates customer service by maintaining a public website (www.mmrrc.org) with access to a searchable repository catalog, an ordering and submission system, centralized customer service, and scientific and technical support. Staff of the ICSC are knowledgeable about all aspects of MMRRC products and services. Researchers can communicate with customer service staff through dedicated MMRRC telephone numbers (800–910-2291 [toll-free North America], 530–757-5710 [international]), email addresses (service@mmrrc.org, support@mmrrc.org), and a service request tracking system. Standard customer operating procedures, including templates and institutional tracking of intellectual property documentation in electronic formats, support both submitters and requesters and their affiliated institutes and facilitate seamless coordination between all Centers in the MMRRC Consortium.

The MMRRC helps to troubleshoot issues raised by customers and attempts to address issues fully. For example, the MMRRC ICSC answers questions about the application submission process and ordering mice or services. Individual MMRRC Centers will respond to customer queries about genotyping results and protocols, recovering and using cryopreserved materials, shipping delays, and mice with an unexpected genotype. In the latter case, the ICSC will help customers find and use the correct stock number if an order was placed incorrectly or contact the MMRRC fulfillment center to review an order if placed correctly. The MMRRC Center will optimize the protocol, select alternate reagents, or design different PCR primers for customers having difficulty with genotype confirmation of mice. If there is discordance between a strain’s phenotype as described on the MMRRC website and the observations made by a customer, the Center will discuss the nature of the discrepancy with the customer and offer to conduct additional analysis as appropriate. Center staff will communicate findings and recommend alternative experimental approaches for using the mouse to meet the research objectives of the customer. During this time the strain might be made unavailable for further distribution until the issue is resolved or removed from distribution entirely if warranted.

## Outreach and education

Although a consortium of four individual Centers, the MMRRC promotes a unified identity to facilitate understanding and awareness of the role of the MMRRC across the research community. This includes unified branding across all outreach instruments, such as the MMRRC exhibit booth, which is deployed at scientific meetings, print (flyers and brochures) and social (X, Linked-in, Facebook) media, and an online web presence (www.mmrrc.org). Materials, including buttons, stuffed mice, bookmarks, pens, video tutorials and other marketing collateral are distributed at scientific conferences and in outgoing shipments of mice, ES cells, and tissues to build long-term memory of MMRRC services. The ICSC regularly posts updates online and on listservs regarding the availability of strains at each Center.

The MMRRC continuously develops innovative marketing methods to build awareness of the mission and goals of the consortium, mouse repository holdings, and benefits for researchers. For example, the utilization of MMRRC mouse strains relies entirely on raising awareness about their availability, variety of phenotypes, and disease relevance. To do so, the MMRRC screens publication databases to correlate the research interests of individual scientists with MMRRC mouse models. This information is used to craft customized emails to inform researchers of specific mice and services relevant to their research. The same system is used to solicit the submission of new mouse models. Research publications highlighting MMRRC mice are frequently publicized in MMRRC news articles at www.mmrrc.org.

## Leveraging complementary organizations

The MMRRC Program actively pursues opportunities to engage and interact with similar and complementary resources and entities. For example, the ICSC offers openly available, automatic updates of mouse holdings at each Center through automatic feeds of data from major biomedical information resources. These include PubMed (pubmed.ncbi.nlm.nih.gov), the Online Mendelian Inheritance of Man (OMIM, www.omin.org), Mouse Genome Informatics (MGI, www.informatics.jax.org), the International Mouse Phenotyping Consortium (IMPC, www.mousephenotype.org), and the Mouse Metabolic Phenotyping Center Live Consortium (MMPC *Live*, www.mmpc.org). Information about the availability of mouse strains can be found online from the MMRRC catalog (www.mmrrc.org/catalog) and from the International Mouse Strain Resource (IMSR, www.findmice.org). Together with the MMRRC, these organizations represent a large share of commonly used entry points for identification of mouse models. For example, all Centers participate in the archiving and distribution of knockout mouse models generated by the NIH Knockout Mouse Production and Phenotyping (KOMP2) Project (commonfund.nih.gov/komp2) (Bradley et al. 2012; Birling et al. 2021). In coordination with its global partners, KOMP2 is working to produce and phenotype mouse strains of male and female mice expressing null alleles for every human orthologous gene in the mouse genome. The MMRRC is now the primary archive and distribution repository for all KOMP2 products, including mice, germplasm, ES cells, targeting vectors, and tissue samples. Investigators seek out these popular knockout mouse strains available at a nominal cost, which is much simpler and faster than making them again in their own laboratories.

In addition, the MMRRC actively integrates with resources and providers of data from other animal species to promote scientific advances benefiting human health. For example, the MMRRC Program shares its mouse phenotype and genotype data with the MONARCH Initiative (www.monarchinitiative.org) (McMurry et al. 2016) to enable semantically integrated computational analysis across mouse and other research animal species that improves understanding the pathophysiology and genetics of human disease. Recently, MONARCH has used data from the MMRRC to further expand its open ontologies, semantic data models, and knowledge graphs for translational research (Putman et al 2024). These activities are essential to maximize the application of knowledge gained from studying mouse models to inform and improve human health.

## Technology transfer

The MMRRC has developed online Conditions of Use (COU) and Material Transfer Agreement (MTA) forms to transfer mouse stocks into and out of the repositories. These documents have been developed after input from the community, the MMRRC’s institutional officials, and NIH technology transfer officers. The paperless COU and Submitter MTA are designed to be time-efficient and eco-friendly. The Submitter MTA can be printed for wet signatures if electronic signatures are not acceptable to a submitter’s institution. All documents must be signed by an authorized Technology Transfer representative from the requester or submitter institution, and where applicable counter-signed by a designated Center official before an order or submission, respectively, is processed. The MMRRC MTA also provides an option for distribution that facilitates the execution of licenses to for-profit entities for strains with commercial value which benefits donors and donor institutions.

## Rigor, reproducibility, and transparency

Although reproducibility of experimental studies using mice has improved significantly (Perrin 2014), centralized mouse repository systems remain a critical and essential component to ensure the reliability of mouse models for biomedical research. The MMRRC is a crucial component of the NIH’s initiative to optimize rigor, reproducibility, and transparency in biomedical research that uses mouse models (Lloyd 2015). For this reason, the MMRRC is committed to upholding the highest experimental design and QC standards that optimize the reproducibility of research studies using mutant mice. For example, understanding, documenting, and accurately reporting the genetic backgrounds of mouse models is essential for recreating an experimental study and achieving reproducible results. Specific QA/QC testing is in place at all Centers. MMRRC mice are annotated with well-documented genetic backgrounds and maintained in specific-pathogen-free (SPF) vivaria. Specifically, the MMRRC helps investigators address NIH’s focus on rigor and transparency by emphasizing:

•*Scientific Premise* – the MMRRC provides highly annotated, genetically engineered mouse models for virtually every field of biomedical research ranging from neurobiology to cancer to infectious disease

•*Rigorous Experimental Design* – the MMRRC provides authentic resources that are easily reported and described using unique identifiers (e.g., RRID numbers) to ensure full transparency and support reproducibility.

•*Consideration of Relevant Biological Variables* – each mouse model is genetically defined (including background strain genetics, incipient congenic strains, and congenic strains) and confirmed SPF. Moreover, the MMRRC has extensive expertise in troubleshooting how other biological variables, such as husbandry factors, microbiota, and sex, may modulate phenotypes. By obtaining mice from the MMRRC, investigators eliminate the risk of genetic drift or contamination with adventitious pathogens that potentially can arise using long-standing in-house colonies or obtaining mice from colleagues. In addition, the MMRRC supports the NC3R’s ARRIVE (Animal Research: Reporting of In Vivo Experiments, arriveguidelines.org) initiative to improve the reporting of research using animals (Kilkenny et al. 2012). The MMRRC stands behind and supports efforts with 3R development, training, and advances and adheres to standardized reporting guidelines.

•*Authentication of Mice Using the Strain Detail Sheet (SDS)* – details about each mouse strain are documented, verified, and authenticated on its unique SDS. The SDS contains information provided by the submitting investigator and curated by MMRRC staff, genotyping protocols, husbandry, care, and management details, and information on availability and prices, including fees for shipping and handling. For example, the SDS will show results from target allele validation and genetic background estimation using the MiniMUGA high-density SNP genotyping array developed by researchers at the MMRRC-UNC (Sigmon *et al* 2020). Strains can be ordered directly from the SDS.

•*Advancing the 3 R’s and Rigor and Reproducibility*–The MMRRC Program is at the forefront of developing and implementing novel broad-based strategies to optimize rigor and reproducibility of research using mouse models. First and foremost, all research use of mice is first reviewed and approved by the Institutional Animal Care and Use Committees at each of the individual MMRRC Centers importing, breeding, archiving, and distributing mutant mouse colonies. Further, the Centers incorporate best practices and standards for the use of mice in all facets of operations and provides guidelines for users on its website, including ARRIVE 2.0 (Percie du Sert *et al* 2020) FAIR (Wilkinson *et al* 2016), PREPARE (Smith et al 2018), and LAG-R (Teboul *et al* 2024). Two key examples are 1) the development, refinement, and deployment of MiniMUGA (Sigmon *et al* 2020) for the assessment and confirmation of background strain/substrain of mutant mice and 2) investment in studies designed to understand the role of microbiota on model phenotypes and to develop tools that aid investigators in considering microbiota in model reproducibility troubleshooting and optimization (Ericsson and Franklin 2021). Centers in the MMRRC Consortium have adopted the highest quality control standards to ensure the fidelity and reliability of mouse models they distribute. As stated previously, all incoming strains undergo verification and characterization including a thorough physical exam by trained technicians noting morphological observations, gene-specific genotyping of DNA extracted from tail snips, background strain analysis using MiniMUGA genome-wide genotyping, metagenomic sequencing, and pathogen surveillance via microbiological culture, PCR, and examination for parasites. Breeding performance, fertility, viability, and sexual dimorphism are noted and recorded while verifying the recoverability of newly cryopreserved germplasm. If not already available, a newly deposited mouse strain is assigned a unique RRID number and listed with its full descriptive characteristics, use protocols, and available formats in the online catalog, linked to scientific references and associated databases. Upon request, a Center will attempt to recapitulate research-relevant phenotypes indicated by the submitting investigator and, importantly, reveal any new or previously unknown phenotypes. When requested as live mice, strains are rederived and maintained in dedicated vivaria with strict pathogen control systems in place to prevent contamination by adventitious pathogens. These and other practices are consistent with 3Rs principles to reduce and refine the number of mice used in research (Tannenbaum and Bennett 2015).

## Disaster planning to preserve and protect from catastrophic loss

Considering the steep investment over time, the scientific and unique value of the resource, and operating principles that minimize duplicative effort and redundant spending to maintain mutant mouse strains for distribution at more than one site, Centers in the MMRRC Consortium have made strategic investments to minimize losses in the event of unforeseen disasters, such as loss of power, fire, flooding, or data breach. For example, Centers split their cryoarchives between different tanks and geographic sites, including at the National Animal Germplasm Program (NAGP) which is a part of the National Laboratory for Genetic Resources Preservation (NLGRP) located in Fort Collins, Colorado, USA (Kaplan 2016, Agca 2017). The electrical power supplying the vivaria, storage tanks, and data servers is provided with full 100% backup using dedicated electrical generators. Fire suppression systems are in place and monitored 24/7/365 by host institution security services, and systems are regularly tested to verify functionality. All data servers are monitored by online cloud services and monthly backups (e.g., tape) are made and stored at distant secure sites for complete data restoration if needed. These and other security practices ensure the preservation of the resource and are shared between all Centers in the MMRRC Consortium.

## Resource sharing plan

The MMRRC Program was established to implement the NIH Sharing Plans for Resources and Data (www.nih.gov). Its prime directive is to encourage the submission and deposition of unique mutant mouse resources developed through NIH-sponsored research into the MMRRC Program, from where they are made readily available to scientists qualified and approved by their institutions to use mice for research purposes. The ICSC makes all mouse resources, genotype, health, husbandry information, phenotype, and other relevant data (including meta-analysis) available on the SDS. Submitted data is confirmed with relevant data and terminology standards. All data is made publicly available after curation, assignment of an RRID number, and listing the strain in the MMRRC online catalog, usually within 30 days of receipt of information and mice from the submitting investigator. The ICSC provides investigators with standard language to include in publications that identify where mutant mice and data are available, how to access data on the MMRRC Program website (www.mmrrc.org), and how to acknowledge the MMRRC Program and NIH funding source.

## Global cooperation and collaboration

While critically important for the US biomedical research community studying mouse models, the MMRRC also serves a worldwide clientele who submit and request mouse strains and services from the repositories. In addition, the MMRRC was a founding member of the original Federation of International Mouse Resources (FIMRe) which has led to closer contacts and coordination of archive and distribution activities with several repositories around the world, including the Japanese Institute of Physical and Chemical Research – BioResource Research Center (RIKEN-BRC), the European Mouse Mutant Archive (EMMA), GemPharmatech (GPT), the Canadian Mouse Mutant Repository (CMMR), the Center for Animal Resources and Development (CARD), and several others (Davisson 2006). This global effort aims to facilitate researchers from around the world access to mouse strains for scientific studies.

## The future of the MMRRC program

The MMRRC Program will continue its active leadership and engagement as the leading public mouse repository system in the US. This includes continuing emphasis on providing research scientists with the most appropriate, valid, and reliable mutant mouse models and services using next-generation technologies (e.g., CRISPR) for well justified, scientifically sound, and reproducible research.

## Data Availability

No datasets were generated or analysed during the current study.
